# Repertory of the Back

**Published:** 1898-05

**Authors:** E. H. Wilsey

**Affiliations:** Parkersburg, W. Va.


### REPERTORY OF THE BACK.

E. H. Wilsey, M. D., Parkersburg, W. Va.

(Continued from April, page 158.)

SPINE.

**Abdomen,** cutting pains, extending in a circle from spine to abdomen, | Aco.
— stitches from spine through upper abdomen to pit of stomach, Thuja.
— violent stitches in spine and in abdomen immediately after supper, Zinc.
**Abscess** of spine, | Sul., | Sil., | Phos-ac., | Hepar., Asaf., Bell., Mez., Iod.
— cold, Sil., Sul., Calc-a., Calc-ph., Calc-iod., Iod., Nat-m., Phos.
— near lumbar vertebra, | Calc-ph.
**Aching** in spine, | Carbo-v., Lyss., Agar., Merc., Kobalt.
— constant in centre of spine, | Sil.
— pain from lower part of spine upward, with stiffness, worse in damp weather, | Nux-m.
— in spine, with sore throat on right side, Lyss.
— periosteal aching down spine, Merc.

### SPINE.

**Aching**, back aching the whole length of spine, Lac-acid.
— pain in small of back or spine, worse on sitting, better when rising and walking or lying down, Kobalt.
— severe down spine, worse from friction, passing off after rising in morning, Lyc-vir.
— along spine, with burning in hips and scapula, **I** Carbo-v.
— soreness in all the vertebræ ; worse by motion and by pressure on spinous processes, **I** Chel.
— along spine, especially between scapula and in lumbar region, with weakness across loins, Arum-dracont.
— in spine, as if it were too weak to support back when bending, Agari.
— and burning in lumbar spine, **I** Helon.
— violently, long-lasting about post-lumbar vertebra, **I** Zinc.
— in lower part of spine, Ol-jec. (sharp, heavy aching).
— along last dorsal vertebra, Asaf., **I** Ascl-s.
— along first lumbar vertebra, Asaf.
**Affections** of spine, **II** Zinc., **II** Formica-r., **I** Alumin., **I** Dolich., **II** Ign., **I** Mez., **I** Sabad., **I** Sil., Apis., Kali-br., **I** Dulc., **I** Ars., **I** Aur-m., **I** Pic-ac., **I** Phos., **I** Iod.
— with diminished sexual desire, **I** China.
— from sexual excesses, **II** Nux-v.
— with melancholy, after disappointed love, **II** Ign.
— cannot raise herself or cannot bear to be raised up in bed on account of severe pain, **I** Lach.
— rheumatic, **I** Dulc.
— with gressus gallinaceus, **I** Ars., **I** Aur-m.
— from inflammation of vertebræ, **I** Phos.
— with anæsthesia or hyperæsthesia, Plumb.
— involving medulla oblongata, **I** Hydr-ac.
— mercurial cases, limbs feel as if shortened, and pains darting, like electric shocks, Mez.
— in paralysis, **I** Ars.
— after getting wet, **I** Rhus.
— due to torticollis, **I** Nux-v.

SPINE.

**Affections** with priapism, ‖ Pic-ac.
— with gressus vaccinus, ‖ Iod., Sec.
**Air**, least draft of air is felt down spine, Sumb.
— a sense of warm air streaming up back into head, ‖ Ars.
**Alcoholism**, progressive locomotor ataxia from alcoholism, ‖ Nux-v.
**Anemia** of spine, resulting from sexual excesses, with palpitation, impotency, and pain in vertex, Phos-ac.
— in those given to sexual excesses, ‖ Phos.
— with early morning diarrhœa, ‖ Phos.
— of spine, Nat-phos.
— in those who use sewing-machines or are much confined to the house, ‖ Nux-v.
— and constipated bowels, ‖ Nux-v.
— from exhausting diseases, ‖ Kali-ph.
**Aphasia**, with spinal congestion, ‖ Gels.
**Ataxia**, locomotor ataxia, ‖ Gels., ‖ Phos.
— progressive locomotor ataxia, ‖ Nux-v. (from alcoholism).
— early stage of locomotor ataxia, Stram.
**Band**, a purple discolored band, two inches wide, along each side of vertebral column, Chloralum.
— in disease of spinal cord, sensation of a band around body, feeling of a plug in spine, so that any motion of body causes a pain as if the plug was sticking still further into body, a paralyzed feeling of knees, patient is scarcely able to walk, feeling as if knees were bandaged, Anac.
**Beaten**, as if beaten and lame in spine, ‖ Ruta. (like lumbago).
**Bent**, stiffness of back, spine becomes bent, ‖ Carbo-v.
**Belching**, a large portion of spine so sensitive to touch that she cried out, turned pale, and was seized with nausea, belching, and retching, ‖ Atro-sul.
**Bending**, spine pains on bending backward, Calc-c.
**Biting**, and burning in a small place on spine, Agar.
**Bifida**, spina bifida, Arn., Ars., Asaf., Bell., Bar-c., ‖ Calc-ph., Calend., Can-s., Carbo-v., Dulc., Eup-perf., Graph., Hepar,

SPINE.

Lach., Lyc., Merc., Mez., Nitr-ac., Phos., | Psor., Ruta., | Sil., Staph., Sul.

**Bifida,** spina bifida of new born, | Calc-c., | Calc-ph.

**Blowing,** feeling as if some one were blowing on spine, Tabac.

**Boring** in spinous processes, Brom.

— sticking in region of spine just below shoulders, Hell.

— burning and sticking in spinal cord, then boring and sticking between scapula, better from motion, Mag-m.

**Break,** sensation as if spine would break with anterior convexity, Kalmia.

— pain in spine, as if it would break, Coccul.

**Breathing,** sticking in right of scapula in morning on breathing deeply, Nat-phos.

— sticking in middle of spine on breathing, Dulc.

**Broken,** tired when walking, so that the lower part of the spine feels broken, Sepia.

— spine feels as if broken when lying on back; in evening when lying on side, Cina.

**Bruised** feeling in spine, Sabad., Spig.

— pain in spine at night, Mag-m.

— pain at top of spine, Plant.

— pain in spine, Ratan.

— pain toward morning, better by rising and by motion, Ratan.

— drawing in spine, frequently taking away breath, Ruta.

— pain above left hip, close to spine, Dulc.

— severe pain in whole spinal column in morning on waking, while lying on his back, Mag-m.

— feeling in spine, severe during rest, Spig.

— pain in spinal muscles, Sul.

— pain in spine when walking, then drawing pressure, as if bruised; better from pressure, Ver-a.

— feeling in lumbar spine, Rhus-t., | Ruta, Hepar.

— feeling in dorsal spine, Arn.

— feeling in cervical spine, Arn.

**Bruises,** ill effects of bruises and shocks to spine, | Con.

### SPINE.

**Burning** in spine, | Agar., Acon., Bell., | Phos., | Ars., Plumb., Ver-a., | Helon., Zinc., | Lyco., Sep.
— along spine, Ver-a.
— pain in spine, | Phos.
— tearing between spine and scapula, Zinc.
— along whole spine, worse on sitting, | Zinc.
— tearing on right side by spine, above sacrum, | Kali-c.
— pain in dorsal spine, Copaiva.
— constant pain in spine, sometimes worse in lumbar region, with great heat and burning, | Kalmia.
— in spine four inches above small of back, Lachn.
— in spots along spine, | Phos.
— along spine, and very great weakness of legs and back, with soreness of muscles and joints, worse from study, | Pic-ac.
— numbness of feet and hands and other parts of body, burning and twingeing sensation referable to spinal column, Physos.
— erosion on the uppermost spinal vertebra, Nat-m.
— superficial burning pains about the dorsal spine, with sudden jerking pains in left ear and cold feeling in small spot in meatus, Chrom-ac.
— pain in spine between shoulders, sore to touch and burning, Ars-m.
— drawing and throbbing pain in spine, Bell.
— pains deep in spine, with violent shooting, | Agar.
— biting in a small place on spine, Agar.
— glow in cerebellum and down spine, Med.
— from spine to head, pain and aching, | Gels.
— up whole spine when lying, | Lyc.
— in spine with myelitis, | Phos.
— in spine with numbness of feet and hands, | Calab.
— along spine with palpitation, | Nat-c.
**Buzzing** in spine, Secale.
**Carbuncle,** small on spine, | Caustic.
**Chest,** sharp sudden stitches beside the spine, darting forward through the chest into cartilages of left ribs in evening, Mez.

SPINE.

**Chest,** pain down entire spine, after constriction in chest, shivering downward, **I** Glon.
— stabbing from third cervical to fifth dorsal vertebræ, striking forward through chest to sternum, worse on motion; inability to straighten spine after stooping, **I** Kali-b.
— rigidity of spinal column in periodic attacks, accompanied by pains through whole chest, Cepa.
— pain on either side of dorsal spine while sitting, similar to that felt in chest, where it is said "the food has lodged," between scapula and lumbar region, **I** Cobalt.
— a slight touch along spine provokes spasmodic pains in chest and indescribable distress in cardiac region, at times heart feels as if twisted, Tarantula.

**Chills,** creeping in spine, Ox-ac.
— creeping along spine, intermingled with hot flashes between shoulder-blades, up back to nape, Polyp.
— and shuddering along spine, during and after menses, **I** Nit-ac.
— rigors after a fit of passion, **I** Hyos.

**Chilliness** down back in spinal region during wet weather, Pic-ac.
— in spine with dull pain in lower cervical and upper dorsal vertebræ, **I** Dulc.
— creeping up spine, **I** Gels.
— of spine with sick headache every month, Polyp.

**Coition,** drawing in spine after coition, Nit-ac.

**Coldness** in spine, **I** Hyos., Ruta.
— in spine, as if cold air were spreading over it, like aura epileptica, Agar.
— in dorsal spine, with shaking, Menyanth.
— in spine during burning and itching of skin, **I** Mez.
— icy coldness through whole length of spine, Thuja.
— downward in spine and through entire abdomen, with nausea and vomiting, **I** Crot-t.

**Concussion,** consequences of spinal concussion, **II** Hyper.

SPINE.

**Concussion**, spine is painful from comfortably driving in a carriage, as from a concussion, Petrol.
**Congestion** of spine, Absin., **|** Art-v., Crotal., **|** Gels.
— in brain and spine, Ver-v.
— state of paralysis of spinal cord, with tetanic spasms, **||** Physos.
— pains in neck, like those of cerebro-spinal congestion, **||** Gels.
— of spinal cord and cerebellum, **|** Gels.
— spinal congestion, with incipient aphasia, **|** Gels.
— softening of spinal cord, congestion or irritation, spinal meningitis, myelitis, or spinal paralysis, Crotal.
— can be used in so-called congestion of spinal cord, Sul.
— worse at base of brain, with throbbing headache, **|** Pic-ac.
**Constriction**, spasmodic, through whole length of spine, especially on motion, Coccul.
**Contractive** sensation in spine, Cham.
— sensation in spine, with vertigo, Sarrac.
**Convulsions**, with jerking of muscles in spinal meningitis, **|** Hyos
**Cough**, dry, hacking, after a fall on back, **|** Hyperi.
— if spine affected in seventh cervical region, he felt depression and paralyzed sensation of forearms and hands, with compression of manubrium sterni, difficulty in swallowing and shrill, paroxysmal cough, Tabac.
— gnawing in spine, with cough, **||** Bell.
**Cracking** in spine, Coccul.
— in cervical region on moving head, **|** Nat-c., Niccol.
— along spine when moving body, Agar.
— noise in vertebræ, Agnus-c.
— in spine on bending head backward, with stiffness of neck, **|** Sul.
**Cramps** in spinal muscles, **|** Helleb.
**Crawling** in spine, as from beetles, **||** Aco.
— sensation of ants crawling along spine, Agar.
**Crawls** in spine, **|** Sal-ac.
— commence at back of neck, moving slowly down, **|** Lach.

SPINE.

**Curvature** of spine, ❙❙ Calc-c., ❙ Calc-ph., ❙ Carbo-v., ❙ Bell., ❙ Fer-iod., ❙ Rhus, ❙❙ Sil., ❙❙ Sul., Syph., ❙ Hepar, ❙ Phos., ❙ Kali-c.

— of spine, occurring after suppression of scabies by ointments, ❙ Sul.

— caries of cervical spine, with great curvature in same region, Syph. (worse at night).

— of spine after vaccination, ❙❙ Thuja.

— of upper part of spine, Puls.

— of spine, with open fontanelles in children, ❙ Puls.

— of dorsal vertebra, ❙ Rhus.

— softening of spine, with curvature, ❙❙ Merc., ❙❙ Sul.

— spinal curvature to the right, painful to touch and on motion, ❙ Sil.

— pain in curved spine, ❙❙ Sil.

— Pott's disease, with curvature, abscesses, emaciation, night sweats, etc., ❙ Sil.

— of spine in a two-year-old child, lasting several weeks, Lyc.

— of spine laterally, ❙ Calc-c., ❙ Merc-c.

— of spine to left, lumbar vertebra bent forward, ❙ Calc-phos.

— of spine in hip disease, ❙ Kali-c.

— of spine in lumbar region, ❙ Bell.

— of spine in lumbo-dorsal region, ❙ Sul.

— of spine, strongly bent to right, ❙ Sul.

— of spine between shoulders, ❙ Hepar.

— of spine when standing, anteriorly in lumbar region, much diminished in a prone position, ❙ Phos.

**Cutting**, from spine to stomach pit, Thuja.

— stitches at ends of right ribs near spine, worse on curving back, Arg-m.

— pain in a circle from spine to abdomen, ❙ Aco.

— pain in lowest region of dorsal spine, ❙ Ascl-s.

— pain in spine, worse on inhalation and moving body, Aug.

— in lumbar spine, worse during stool, Rheum.

**Damp**, distressing sensation of damp clothes on spine, ❙❙ Tuberc.

### SPINE.

**Darting,** like shocks of electricity in mercurial cases (diseases of spine), Mez.

— up spine on stooping, **I** Sil.

**Debility,** great of spine after typhoid fever, Selen.

**Deglutition,** pressure in spine during deglutition, Kali-c.

**Dislocated** feeling in middle of spine, worse by sudden bending to the right, Thuja.

— as if dislocated, painful in cervical vertebra, when lifting arm, **I** Ang.

**Down,** sore pain down cervical vertebra, **I** Ham.

— pain down entire spine, after constriction in chest, shiverings downward, **I** Glon.

— pricking up and down spine, Jug-cin.

— pain down spine, Physos., **I** Coccul.

— numb tired pains up and down spine and in head, **I** Curare.

**Drawing**, tearing down spine, **I** Cina.

— pain at nape of neck and each side of spine and pains in small of back, as if bruised, **I** China.

— down right side of spine from axilla to lowest ribs, with tearing, Guaic.

— along spine and in calves, with weakness in feet, Thuja.

— pain in spine when walking, then drawing pressure as if bruised, better from pressure, Ver-a.

— painful near spine to left, downward from shoulder blade, Bad.

— pressure between right scapula and spinal column, Bell.

— burning and throbbing pain in spine, Bell.

— pain extending from spine into hip joints, Mosch.

— and tension in spine, **I** Nat-m.

— bruised pain in spine frequently taking away breath, Ruta.

— pain in middle of spine, with drawing pains opposite to it on back part of stomach, **I** Stram.

— slight pain along spine in P. M., changes to a seated, dull tearing in joints of legs, worse by walking, Stront.

— in spine extending upward on stooping, Sul.

SPINE.

**Drawing** in spine after coition, Nit-a.
— head drawn to one side owing to spinal disease, | Nux-v.
— and tearing in and near spine, | Cap.
— downward and pain in spinal cord, worse by stooping or bending, Daphne.
— pain in seventh cervical vertebra after a walk, with drawing in nape, Curare.
— in spine like a painful weakness while sitting and stooping, Zinc.
**Dropsy** of spine, | Lyss.
**Dyspnœa,** caused by pressure upon upper three dorsal vertebræ, | Chin-s.
— pressing stitches along spine, mostly in sacrum with dyspnœa, Tarax.
**Electric,** frequent stitch like electric shocks in a zigzag course along spine from lumbar to scapular region, Euonymus.
**Erect,** pain across shoulders and spine, must stoop, cannot walk erect, | Can-ind.
**Erosion**, burning on the uppermost vertebra, Nat-m.
**Eructation**, abortive, with pressure in middle of spine, Zinc.
**Eruption**, petechiæ, in cerebro-spinal disease, | Ver-v.
— several groups of pustules near spine, having a hemorrhagic appearance, spreads from one group to another till it reaches axillary line, | Lach.
— large greenish spots at base of spine, Chloral.
— variola-like pustules, painful and suppurating on spine, | Sil.
**Exhaustion** of spine, following acute diseases, | Pic-ac.
**Exudation** of spine, Arn.
**Fall**, great pain in spine after a fall from a hammock, growing daily more severe, | Hyper.
— dorsal spine feels as if it would fall in after sitting a while, Bar-c.
**Faint** feeling and nausea caused when standing by tired pain in lower part of spine, Sepia.
**Formication** in spine, || Acon., Agar.

### SPINE.

**Formication** and numbness along spine into extremities, | Nux-v.
— as from going to sleep in the spine, Con.
— along the spine, | Ars. (in sexual excess).
— in lower half of spine, | Phos.
**Gnawing** in spine, || Bell. (with cough).
— with sticking in middle of spine, Helleb.
— in spinal cord extending up to neck, evening after lying down, preventing sleep, and causing constant tossing about, Mag-m.
**Gressus** gallinaceus with diseased spine, | Ign., | Aur-mur., | Ars.
— vaccinus with spinal complaint, | Iod., | Calc-c., Secale.
**Heat** in spine, Hyos.
— in cervical spine to occiput, Nitr-sp-d.
— in medulla and spine for a whole week, | Med.
— in lower part of spine, | Pic-ac.
— second and third lumbar vertebræ sensitive to pressure of hot sponge, | Agar.
— progressive spinal paralysis with partial contraction of affected muscles, anæsthesia and increased heat, | Phos.
— and redness down spine, Ver-v.
— warm air streaming up back into head, | Ars.
— in spine, follows sensation as if she were going to faint, Med.
— rising of warmth along spine, | Lyco.
— sensation of heat in spine, Plumb.
— in spine for a week, | Med.
**Heaviness** in spine, | Phos. (Heavy pain in lower spine, Ol-jec.)
— in lumbar vertebra, Ammoniac.
**Hyperæmia** of spinal cord, | Dulc.
**Hyperæsthesia** and great sensitiveness of spine from sixth cervical vertebra to small of back, slightest touch intolerable, | Cup.
— of spinal cord or reflex irritation, | Diosc.
**Hypogastrium,** pain in hypogastrium, extending into spine, Iod.

SPINE.

**Hysterical,** feeling of lightness in body from spinal exhaustion in onanists and hysterical subjects, I Gels.

**Inflammation** of spinal cord, I Calc-c., I Phos.

**Injuries** to spine, I Arn., I Hyper., Ruta., II Con., Sil.

**Injury,** in nervous affection following injury to spine, chronic neuritis, particularly when pressure upon spine causes pain in remote parts, especially head, Sil.

**Irritation** of spine, I Chin-s., Coca., I Coccul., Crotal., I Cup-m., I Hepar, I Naja., I Nat-m., I Nux-v., I Ol-jec., I Phos., I Phos-ac., I Phyto., I Puls., I Ran-b., I Rhus., I Sec., I Therid., I Zinc., II Sil., I Tarent.

— of spine and sensitiveness between vertebræ, I Nat-m.

— of spine with sudden loss of power in legs in the morning, I Nux-v.

— of spine, hands and feet go to sleep easily, I Nux-v.

— of spine, due to sexual excesses or masturbation, I Puls.

— pain depending on spinal irritation, I Ran-b.

— every nerve of spinal origin is irritated by this drug, I Physos.

— of spine, great sensitiveness between vertebræ, sits sideways in chair to avoid pressure against spine, I Therid.

— of spine in beginning of typhoid, Valer.

— of spine with paralytic symptoms, II Sil.

— of spine with amaurosis first right then left eye, I China.

— of spine in painful affections, I Atrop-s.

— of spine, causes cardiac neurosis, I Kali-br.

— of spine, causes cephalagia, I Sil.

— of spine, causes hysteria, I Kali-br., I Therid.

— of spine, with chronic leucorrhœa, Ziz.

— of spine, with desire to lie down, I Nux-v.

— lower part of spine, paralytic incontinence of urine from spinal irritation, II Nux-v.

— could not bear the least noise, jar of foot on floor worse, must cry out, I Therid.

— affecting muscles, causing cough, even spasmodic stricture of œsophagus, I Naja.

SPINE.

**Irritation** of spine causes nymphomania, ❙ Sil.
— pressure of finger between vertebræ causes patient to wince, ❙ Calab.
— of spine, causes prosopalgia, ❙ Ign.
— of spine, with prostration, ❙ Zinc.
— of spine, with dilatation of pupils, ❙ Nux-v.
— of spine of reflex origin, ❙ Diosc.
— of spine, rheumatic, ❙ Bar-c., Caust.
— of spine, with itching in uterus, ❙ Plat.
— of spine, caused by getting wet, ❙ Rhus-t.
**Jerking** in middle of spine, ❙ Cina.
— stitching in middle of spine on walking in open air, better when standing, jerking in sacrum and at same time above ankle, Calc-ac.
— convulsions, with jerking of muscles in spinal meningitis, ❙ Hyos.
— superficial burning pains about the dorsal spine, with sudden jerking pains in left ear and cold feeling in a small spot in meatus, Chromic-ac.
— jerk-like above right hip, near spine, Dulc.
— small violent jerking, sticking in middle of spine, Phos-ac.
— pain in spine partly jerking and partly drawing, becoming firmly seated, worse on upper part of thighs and so impeding walking, Mosch.
**Jerks** along left side of spine in region of ninth rib, Formica-r.
**Knotted** cords from right mammæ to spine, ❙ Asterias.
**Lame,** pain in spine as if lame, Ruta.
**Lancinating,** in upper part of spinal column toward right scapula, Cina.
— from lower dorsal vertebra through chest, arresting breathing, Berb.
**Lie,** peculiar and great weakness on both sides of the spine, she could not lie on back, Apis.

